# Conditions of Registration Applicable during the Period of Grace to Existing Nurses

**Published:** 1916-10-28

**Authors:** 


### THE COLLEGE OF NURSING, LIMITED.

### Conditions of Registration Applicable during the Period of Grace to Existing Nurses.

The following are the latest conditions which have been approved by the Council of the College. It will be seen that they are much fuller than those previously issued, and include important information as to the class of institution which will be recognised for training.

Nurses Trained before 1900.

1. An applicant whose training was completed before December 31, 1899, is required—

(a) To be of good character.

(b) To produce evidence of training to the satisfaction of the Council, followed by at least five years' *bona fide* practice as a nurse.

Nurses Trained after 1900.

2. An applicant whose training was completed after January 1, 1900, is required—

(a) To be at least twenty-one years of age.

(b) To be of good character.

(c) To hold a certificate or certificates of three years' training in a Nurse-Training School, or Schools, recognised by the Council for the purpose of admitting practising nurses to the Register of the College; or

(d) To hold a certificate of not less than two years' training in a Nurse-Training School recognised by the Council for the purpose of admitting practising nurses to the Register, followed by at least two years' *bona fide* practice as a nurse.

3. Certificates of not less than three years' training are required in the case of a nurse trained—

(a) At a recognised Poor-Law infirmary.

(b) At recognised affiliated hospitals.

Institutions Recognised for Training.

4. For the purpose of admitting practising nurses to the Register of the College, general civil hospitals and infirmaries are recognised for training in which—

(a) There is an average number of not fewer than forty beds in daily occupation throughout the year.

(b) There is a resident medical or surgical officer.

(c) There is given at least one course of nursing lectures annually, followed by examination for qualification.

5. For the purpose of admitting practising nurses to the Register of the College, Poor-Law infirmaries are recognised for training in which—

(a) There are not fewer than 250 beds.

(b) There is a resident medical or surgical officer.

(c) There is given at least one course of nursing lectures annually, followed by examination for qualification.

Fee.

The fee for registration and membership has been fixed at £1 1s. (one guinea).

How to Register.

Forms of application for registration and membership can be obtained from the Secretary of the College. The envelope should be marked "Registration," and an addressed and stamped envelope enclosed for reply. Address: The Secretary, the College of Nursing, Limited, 8 Vere Street, Cavendish Square, London, W.

### MEMBERS OF COUNCIL, OCTOBER 1916.

Chairman: The Hon. Arthur Stanley, C.B., M.P.

Miss A. B. Baillie, R.R.C., Matron, Royal Infirmary, Bristol.

Miss E. Barton, R.R.C., Matron, Chelsea Infirmary, S.W.

Mr. J. Cantlie, F.R.C.S.

Miss R. Cox-Davies, R.R.C., Matron, Royal Free Hospital, W.C.

Miss A. C. Gibson, late Matron, The Infirmary, Birmingham.

Miss A. W. Gill, R.R.C., Lady Superintendent, Royal Infirmary, Edinburgh.

Professor Glaister, M.D., Glasgow University.

Miss L. V. Haughton, Matron, Guy's Hospital, S.E.

Miss A. Hughes, General Superintendent, Q.V.J. Nurses.

Miss A. McIntosh, Matron, St. Bartholomew's Hospital, E.C.

Miss J. Melrose, R.R.C., Matron, Royal Infirmary, Glasgow.

Mr. W. Minet.

Miss E. W. Mowat, Matron, Whitechapel Infirmary, E.

Miss E. M. Musson, R.R.C., Matron, Birmingham General Hospital.

Miss M. E. Ray, R.R.C., Matron, King's College Hospital, S.E.

Miss M. E. Sparshott, R.R.C., Lady Superintendent, Royal Infirmary, Manchester.

Miss A. Lloyd Still, Matron, St. Thomas's Hospital, S.E.

Miss S. A. Swift, R.R.C., Matron-in-Chief, Joint War Committee.

Dr. H. G. Turney, F.R.C.P.

Miss C. E. Vincent, R.R.C., Lady Superintendent, Royal Infirmary, Leicester.

Dr. Jane Walker.

*Hon. Treasurer*: Mr. Comyns Berkeley, M.C., M.D., F.R.C.P. *Hon. Secretary*: Sir Cooper Perry, M.D., F.R.C.P. *Secretary*: Mary S. Rundle, R.R.C. *Solicitors*: Messrs. Charles Russell and Co., 37 Norfolk Street, Strand, W.C. *Auditors*: Messrs. Barton, Mayhew and Co., Alderman's House, Alderman's Walk, E.C.

